# Battling the unknown: Using composite vignettes to portray lived experiences of COVID-19 and long-COVID

**DOI:** 10.1371/journal.pone.0284710

**Published:** 2023-04-26

**Authors:** Rachel L. Knight, Kelly A. Mackintosh, Joanne Hudson, James Shelley, Zoe L. Saynor, Melitta A. McNarry

**Affiliations:** 1 Applied Sports, Technology, Exercise and Medicine Research Centre, Faculty of Science and Engineering, Swansea University, Swansea, United Kingdom; 2 School of Sport, Health and Exercise Science, Faculty of Science and Health, University of Portsmouth, Portsmouth, United Kingdom; UNITED STATES

## Abstract

Understanding the day-to-day lived experiences of individuals who have had or are still recovering from Coronavirus Disease-19 (COVID-19), whilst a complex challenge, presents the opportunity to listen and learn. Composite vignettes provide a novel approach to explore and present descriptive portrayals of the most commonly derived experiences and recovery journeys. The thematic analysis of 47 shared accounts (semi-structured interviews with adults aged ≥18 years; 40 females; 6–11 months post-COVID-19 infection) produced a series of four intricate character stories written through the lens of a single individual. Each vignette gives a voice to and captures a different experience trajectory. From the point of initial symptom development onwards, the vignettes depict how COVID-19 has affected everyday lives, focusing on the secondary non-biological socio-psychological effects and implications. The vignettes highlight in participants’ own words: i) the potential negative implications of not addressing the psychological effects of COVID-19; ii) the lack of symptom and recovery linearity; iii) the ongoing ‘lottery’ of access to healthcare services; and iv) the highly variable, yet generally devastating, impacts that COVID-19 and consequent long-COVID has had across multiple facets of daily living.

## Introduction

Coronavirus Disease-2019 (COVID-19) has presented the world with a phenomenon we are still striving to understand and live with. Whilst significant advances have been made regarding acute medical treatments and preventative or symptom-limiting vaccines, there remains limited knowledge and understanding of the complexities associated with an individual’s symptom pattern and recovery journey. Nonetheless, it has become apparent that symptom presentation and recovery experiences are not linear [1]. Specifically, where some individuals develop persistent symptoms, ranging from, but not limited to, ear, nose, and throat, neurological, musculoskeletal, gastrointestinal, dermatological, respiratory, and cardiac symptoms [2], or report relapses even after minimal acute illness, others recover quickly and fully [3].

Residual or new symptoms that persist or develop in individuals post COVID-19 infection, that continue for longer than 12 weeks, have been collectively termed “post-COVID-19 syndrome”, with the secondary term “long-COVID” used to describe both post-COVID-19 syndrome, and ongoing symptomatic COVID-19 where symptoms continue for more than four weeks [2]. The profound impact that not only the symptoms themselves, but also the secondary effects of a COVID-19 diagnosis, are having on individuals both in the short- and long-term is becoming increasingly evident. For example, negative effects have been observed on the ability to engage with any physical activity or undertake activities of daily living as far on as eleven months post-infection, respectively [1].

Listening to, and learning from, the lived experiences of individuals who have had, and are potentially still recovering from, COVID-19 has been highlighted as a priority by the National Institute for Health and Care Research (NIHR) [4]. To date, reported findings from across the world have highlighted that the impact goes beyond biological effects [5]. In Denmark, Missel et al. [5] found that being diagnosed within the first wave of the pandemic often changed the dynamic of social relationships, with individuals (15 confirmed cases of COVID-19) becoming not only recipients of care but a source of curiosity. In China, Liu and Liu [6] discussed the acute post-hospitalization psychological impact in 16 adults in terms of both fears and the uncertainty associated with the virus and managing family responsibilities. Additionally, within South Korea and India, individuals reported experiencing negative attitudes from outside their social and familiar circle that resulted in social stigmatization [7–9].

The purpose of this study was to explore and present the day-to-day lived experiences of adults in the United Kingdom (UK) who contracted COVID-19 during the early stages of the pandemic, namely between February and May 2020. The primary focus was the secondary socio-psychological effects and implications. Using a novel composite vignette approach [10], descriptive portrayals of the most common themes and experiences derived from the analysis of shared accounts have been used to produce a series of intricate character stories. Each vignette seeks to give a voice to a different experience trajectory, whilst capturing from the acute onset of symptoms through the ongoing recovery process (6–11 months post-infection), how COVID-19 and subsequent long-COVID has shaped the individuals’ everyday lives [11].

## Methods

### Philosophical framework

It is acknowledged that, traditionally, the exploration of individual experiences through narrative analysis is situated within a social constructivist epistemology, whereby knowledge is viewed as being constructed through the different meanings that individuals attribute to their specific experience [12]. However, the data analysis within this study was driven by a post-positivistic position viewed through a critical realist lens. While this approach is underpinned by the need to seek a reality as close to the truth as possible, it is accepted that this can never be definitive, and is affected by social parameters and the specific context in which it is situated [13]. Given the unique and variable situation that COVID-19 has presented, and the current limited insight into this phenomenon, this study was approached with the reality that it was more than likely that multiple “character experiences*”* would be outlined. However, through the data analysis processes employed, in line with post-positivistic principles, the most prevalent individual and patterns of experiences were subsequently identified and outlined.

### Participants

To participate, eligibility criteria mandated that individuals were aged ≥18 years and had previously been infected with COVID-19. Due to inconsistent testing availability in the UK at the start of the pandemic, a positive pathological test was not mandatory. Exclusion criteria comprised any pre-existing condition that could limit an individual’s capacity to exercise (e.g., cardiovascular disease) or a lack of capacity to understand the study protocol [14]. Overall, 47 adults (40 female), 6–11 months post-COVID-19 infection consented to participate.

### Procedures

Ethics approval was granted by the Health Research Authority, Health and Care Research Wales (20/HR/3536) and local University Research Ethics Committee. Participants were recruited from the UK via social media and University webpages as part of a larger randomized control trial (n = 281) investigating the use of inspiratory muscle training [14] and were invited to take part in both the wider intervention, and the qualitative interviews, until a target of 50 pre-intervention interviews was achieved. A total of 47 participants were included following withdrawals from the study. Prior to the commencement of data collection, fully informed verbal consent was obtained with further written consent returned following study cessation. The study is reported according to the Standards for Reporting Qualitative Research: A synthesis of recommendations (SRQR) checklist [15] (S1 File).

## Data collection

Data for this study were collected via online semi-structured interviews with participants. Each interview, lasting between 20 and 76 minutes, was conducted by a trained researcher (JS), recorded via online video conferencing software (Zoom Video Communications, San Jose, CA), and transcribed verbatim. The interview guide used in this study, devised by authors MAM and KAM and subsequently updated by JS and JH, was designed to explore participants’ acute and ongoing experiences of COVID-19, specifically discussing their general experiences, symptoms, effects on day-to-day life and lifestyle, and their approaches to recovery.

### Data analysis

**Fig 1:**
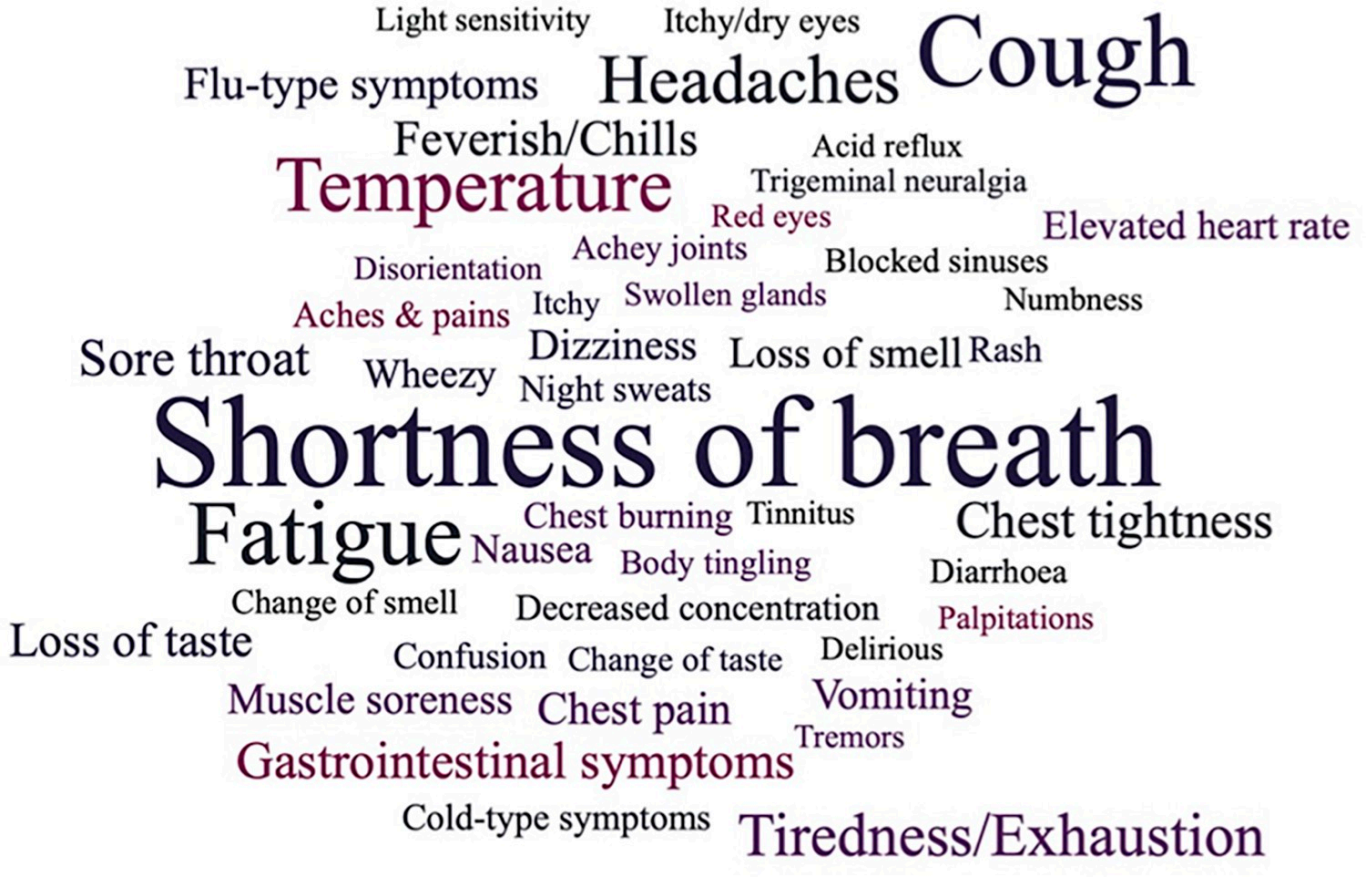
Word cloud illustrating the frequency and array of different symptoms reported within the acute phase of participant experiences.

**Fig 2:**
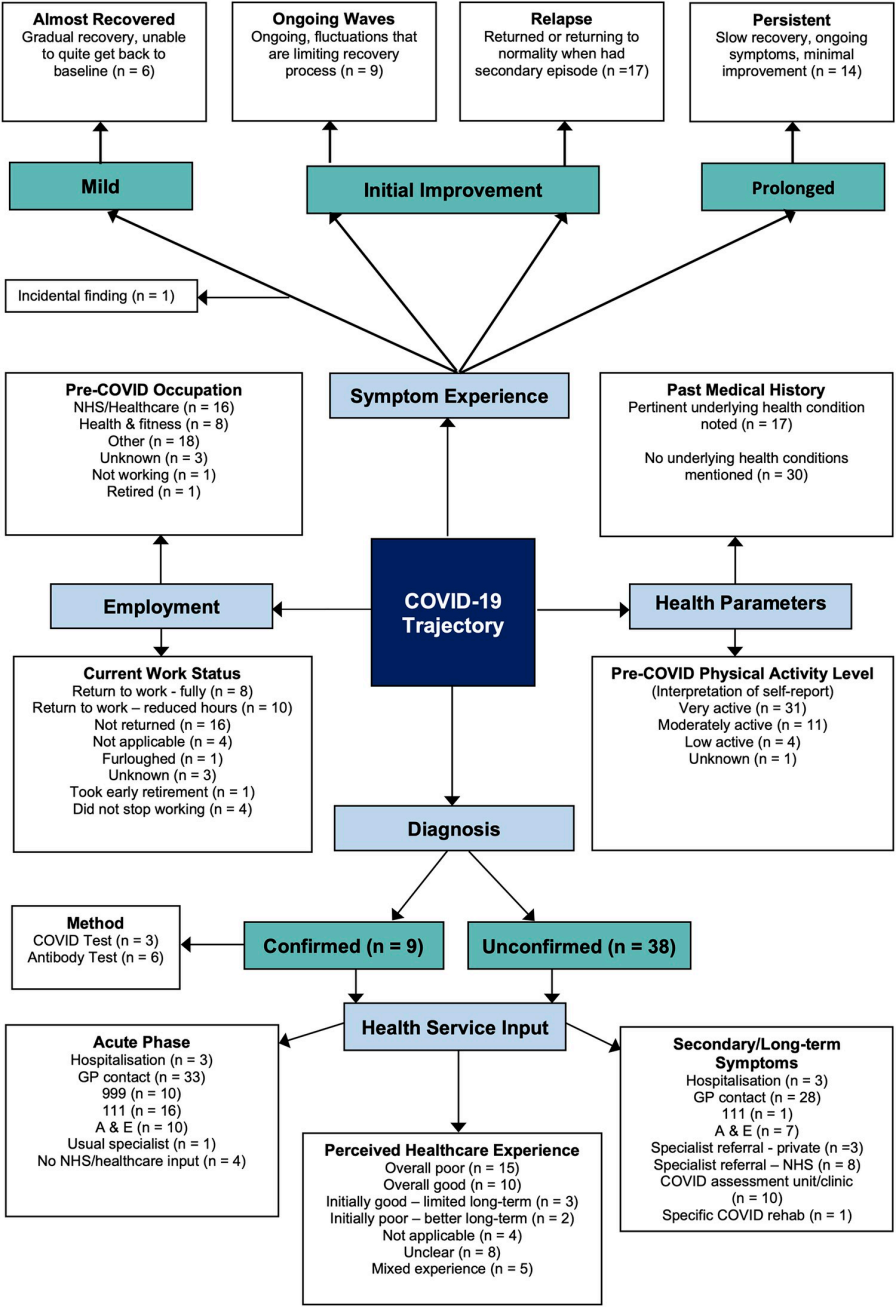
Pen profile of participant characteristics and experience patterns. ^*a*^ A & E = Accident and Emergency; COVID = coronavirus disease; GP = General Practitioner; n = number; NHS = National Health Service. ^*b*^ Full vignette titles: ‘Almost recovered—out of sight, out of mind, but I just had to get on with it’; ‘Ongoing waves—trapped on the COVID roller-coaster, when the horizon just keeps falling out of sight’; ‘Relapse—recover, relief, relapse, battling the unknown enemy’; and ‘Persistent—life-changing and lifelong…is this it now?’.

Rigorous data analysis and interpretation processes were implemented and followed. Given the paucity of existing research regarding this phenomenon, an inductive thematic analysis based only on the semantic meaning of the data was used [16]. Primary in vivo and descriptive transcript coding was undertaken by one author (RLK), who, having read each transcript multiple times, coded and summarized participant experiences, identified the key time-point experiences within each individual’s COVID-19 journey, and started to form connections between the transcripts [17]. Secondary content coding was separately used to categorize key time-point experiences. A frequency count of acute symptoms reported was also recorded. These findings were then used to set the context of the collective participant experiences by forming a word cloud that illustrates the frequency and array of different symptoms reported within the acute phase of participant experiences (Fig 1), and a pen profile of participant characteristics and experience patterns to visualize and cluster the core themes that evolved (Fig 2). Additional participant demographic data and a breakdown of the characteristics and experiences by theme is provided in Table 1. It is pertinent to note that specific questions were not asked regarding the reported participant characteristics; counts are purely based on the content coding. Therefore, it is possible that where, for example, none were recorded, the participant did not refer to this characteristic during the course of the interview.

**Table 1:** Participant demographic data, and breakdown of characteristics and experiences by theme.

		Almost Recovered (n = 6)	Ongoing Waves (n = 9)	Relapse (n = 17)	Persistent (n = 14)
**Demographics**	**Female**	4 (67%)	7 (78%)	15 (88%)	14 (100%)
**Pre-COVID occupation**	**NHS/healthcare worker**	3 (50%)	3 (33%)	4 (24%)	5 (36%)
	**Health & fitness industry worker**	0	2 (22%)	3 (18%)	3 (21%)
	**Other**	2 (33%)	4 (44%)	8 (47%)	4 (29%)
	**Unknown**	0	0	2 (12%)	1 (7%)
	**Not working**	0	0	0	1 (7%)
	**Retired**	1 (17%)	0	0	0
**Current work status**	**Returned to work—fully**	4 (67%)	1 (11%)	2 (12%)	1 (14%)
	**Returned to work—reduced hours**	0	2 (22%)	4 (24%)	4 (29%)
**Current work status**	**Not applicable**	1 (17%)	2 (22%)	0	1 (7%)
	**Not returned**	0	2 (22%)	8 (47%)	6 (43%)
	**Furloughed**	0	0	0	1 (7%)
	**Unknown**	0	1 (11%)	1 (6%)	1 (7%)
	**Took early retirement**	0	0	1 (6%)	0
	**Didn’t stop work**	1 (17%)	1 (11%)	1 (6%)	0
**Diagnosis**	**Confirmed**	1 (17%)	1 (11%)	3 (18%)	3 (31%)
	**Unconfirmed**	5	8 (89%)	14 (82%)	11 (79%)
**Health service input acute phase**	**Hospitalisation**	0	0	2 (12%)	1 (7%)
	**GP contact**	1 (17%)	9 (100%)	12 (71%)	11 (79%)
	**999**	0	0	6 (35%)	4 (29%)
	**111**	2 (33%)	2 (22%)	6 (35%)	6 (43%)
	**A&E**	0	2 (22%)	6 (35%)	2 (14%)
	**No NHS/healthcare input**	3 (50%)	0	0	0
	**Usual specialist**	0	0	0	1 (7%)
**Health service input secondary/long-term symptoms**	**Hospitalisation**	0	0	3 (18%)	0
	**GP contact**	1 (17%)	4 (44%)	13 (76%)	10 (71%)
	**111**	0	0	0	1 (7%)
	**A&E**	0	0	4 (24%)	3 (21%)
	**Specialist referral (Private)**	0	1 (11%)	2 (12%)	0
	**Specialist referral (NHS)**	0	1 (11%)	4 (24%)	3 (21%)
	**COVID assessment unit/clinic**	1 (17%)	3 (33%)	4 (24%)	2 (14%)
	**Specific COVID rehab**	0	0	1 (6%)	0
**Perceived healthcare experience**	**Overall poor healthcare experience**	2 (33%)	3 (33%)	7 (41%)	3 (21%)
	**Overall good healthcare experience**	1 (17%)	3 (33%)	3 (18%)	3 (21%)
	**Initially good—limited long-term**	0	0	2 (24%)	1 (7%)
	**Initially poor—better long-term**	0	0	2 (24%)	0
	**Not applicable**	3 (50%)	0	0	0
	**Unclear**	0	2	2 (24%)	4 (29%)
	**Mixed experience**	0	1 (11%)	1 (6%)	3 (21%)
**Past medical history**	**Noted pertinent underlying health condition**	0	3 (33%)	7 (41%)	7 (50%)
	**Nil noted**	6 (100%)	6 (67%)	10 (59%)	7 (50%)
**Pre-COVID physical activity level (interpretation of self-report)**	**Very active**	4 (67%)	4 (44%)	14 (82%)	8 (57%)
	**Moderately active**	2	4 (44%)	3 (18%)	2 (14%)
	**Low active**	0	1 (11%)	0	3 (21%)
	**Unknown**	0	0	0	1 (7%)

^a^ Breakdown for n = 1 participant ‘incidental finding’ not provided to maintain anonymity

^b^ A & E = accident and emergency; COVID = coronavirus disease; n = number; NHS = National Health Service; SD = standard deviation; 999 = emergency services phone number; 111 = non-emergency medical assistance and advice phone number.

^c^ Full vignette titles: ‘Almost recovered—out of sight, out of mind, but I just had to get on with it’; ‘Ongoing waves—trapped on the COVID roller-coaster, when the horizon just keeps falling out of sight’; ‘Relapse—recover, relief, relapse, battling the unknown enemy’; and ‘Persistent—life-changing and lifelong…is this it now?’.

Pen profiles have been identified as a clear way for researchers with both quantitative and qualitative backgrounds to present analysis outcomes [18]. Using this approach also ensured that whilst the aim was to establish the most prominent COVID-19 experiences, all themes were given an equal chance of identification and representation. On the completion of this stage, JH and JS cross-checked the transcripts against each generated journey to ensure the accuracy of generated experience themes/consistency in approach. All authors collaboratively discussed the generated themes, and RLK subsequently formed these into the core character experiences to be represented using a form of creative non-fiction, composite vignettes [10].

Presenting the lived experiences of individuals through the fusion of data created from uniquely shared accounts is a novel way to represent the key, yet often intricate, findings of qualitative data [19]. Composite vignettes descriptively portray the generated data as indirect character stories constructed from details contrived by the authors, led by the common themes, and illustrated with direct participant quotations [17]. In this instance, the vignettes highlight the journey from the acute onset of symptoms, through the ongoing recovery processes (6–11 months post-infection), detailing changes, emotions, contributary factors, and adopted approaches to recovery. Whilst, as previously identified, creative non-fiction is not normally aligned with the outlined philosophical assumptions underpinning the data analysis within this study, the overall stance of the research team varied along the philosophical continuum, and it was felt that this approach was the most fitting for ‘three’ key reasons. First, given the unique situation that arose from the COVID-19 pandemic, whilst we may not be able to identify a definitive truth regarding individuals’ experiences, understanding the most prevalent of these will help guide future health strategies and policies. Second, this approach allows us to consider and present, from data that were systematically collected [19], the individual’s whole journey to date, not just its component parts. Third, this approach provides an accessible form of data presentation, which should be understandable to a wider audience [20]. Given the public interest and investment in the COVID-19 pandemic, it is important to present findings in a way that is interpretable and accessible to all who wish to engage with them.

Following agreement on the outline of each of the four composite vignettes, the transcripts pertaining to each one were revisited by RLK and direct quotations from the coded transcripts, representative of each theme and aligned to each character, were compiled. These quotations were subsequently used as the basic structure for each vignette to ensure that they accurately represented the experiences they intended to portray. Following the initial drafting by the first author (RLK), each vignette received extensive feedback from the other authors and was revised accordingly until deemed to be an accurate and complete representation of the data.

### Criteria for judgement and reflexivity

It is pertinent to note that the author who undertook the primary data analysis is a practicing healthcare professional. However, they were not actively involved in the care of individuals with COVID-19 during the pandemic and did not conduct the interviews. Moreover, the participants were volunteers who were part of a wider trial seeking to improve recovery; therefore, they were potentially more likely to have had ongoing symptoms. During the analysis processes and the construction of the vignettes, the research team regularly acknowledged and reflected, both in isolation and collectively, on their own biases. For example, those who recruited the participants, undertook the interviews, or were involved in data collection for the parent study, often had a greater recollection of certain experiences. The initial data analysis and vignette construction by a researcher (RLK) who had no contact with the participants minimized the influence of such biases on data interpretation, synthesis, and presentation. To ensure credibility, transparency, rigor, and quality control [21], JH (who also had no contact with the participants) and JS acted as ‘critical friends’ to RLK. Given our philosophical position, our approach to rigor and quality was in accord with previous research, rejecting a criteriologist perspective which suggests that the use of certain methods will inevitably enhance rigor (e.g., member checking; see Day et al. [22]). Smith and McGannon [20] present a detailed explication of this approach. A transparent data analysis audit trail was maintained throughout the whole process by RLK. An exemplar of the data synthesis process used to create the vignettes is provided in S2 File.

## Results

Whilst representing the COVID-19 experiences of multiple participants, the following vignettes are written as fictional composites through the lens of a single individual. Based on the emergent themes, four vignettes are presented: ‘Almost recovered—out of sight, out of mind, but I just had to get on with it’; ‘Ongoing waves—trapped on the COVID roller-coaster, when the horizon just keeps falling out of sight’; ‘Relapse—recover, relief, relapse, battling the unknown enemy;’ and ‘Persistent—life-changing and lifelong… is this it now?’. Direct quotations are identified in *italics* within the text.

### Almost recovered—out of sight, out of mind, but I just had to get on with it

“My symptoms started before the first lockdown in the UK, in March 2020. There wasn’t so much known about it then, so initially *I didn’t think it could be COVID*; I didn’t have the exact pattern of symptoms that were being discussed and they were quite mild compared to what was being reported in the media. I think I’ve got off quite lightly compared to some people. I was *pretty much in bed completely for the first nine days*, but after that I’ve gradually, thankfully, been able to nearly return to normal. Don’t get me wrong, it’s still been a struggle at times, *it was a real stopper in quite a lot of activities* to start with, and not being able to quite get over that last hurdle, and back to my pre-COVID baseline physical activity levels is really *frustrating*. I don’t know if some of it is me holding back, but part of me is frightened to push any further in case I cause more damage. There is definitely *a little bit more trepidation*. *The problem we’ve got is*, *nobody knows* what the long-term health implications could be, and that is really worrying. It also doesn’t help that we have been pretty much left to figure out how to recover by ourselves, *wait it out*, *see if you get better*. At the time, it felt like unless you were so unwell you needed to go to hospital, nobody did anything, nobody cared, *I had to just get on with it*.”

### Ongoing waves—trapped on the COVID roller-coaster, when the horizon just keeps falling out of sight

“For the past eight months, my life has been one long roller-coaster ride. My symptoms developed over a period of a few weeks, and after feeling like they were attacking every system in my body, did start to improve. The problem is, every time I thought I was making progress with my recovery, *periodically all these symptoms just came hurtling back*. The unpredictability of these fluctuations was difficult to deal with, I was *sort of trapped in this situation*. Most of the time, the flare-ups, exacerbations, or whatever you want to call them, were linked to physical exertion. It is so hard trying to work out what you can do without *paying the price afterwards*, especially when trying to manage the *breathing difficulty* and *fatigue*. It all seemed to come down to pacing, planning, and prioritization–deciding what is the most important thing you need to do and how you can get it done, but it’s hard to pace when you feel fine at the time. *The pacing thing has been quite crucial*, even if it wasn’t that easy to initially master. I did find myself becoming risk-averse though, trying to avoid things that *might trigger me being really ill*. I don’t think you can help being nervous or anxious in this type of situation, when you never quite know how your body will respond. It’s also difficult to explain to other people–your family, your General Practitioner (GP), your boss. No confirmed diagnosis, no explanation for your symptoms… one day you’re fine, one day you’re not. *That is the killer really*. *You just feel you’re missing out on life*, but the good days are slowly starting to outnumber the bad.”

### Relapse—recover, relief, relapse, battling the unknown enemy

*“I kind of thought ‘oh I’ve dodged a bullet’* when my initial symptoms of what was presumed to be COVID resolved enough for me to return to work and start to build up my physical activity again. Then, after about a week of feeling ‘normal’, bang, there it was again, and this time it seemed worse than before. It was at that point the battle began. I had avoided the healthcare system at the beginning because we were not being directed there, and to be honest I didn’t think I needed it. But this time, frequent calls to the GP, and a few panicked visits to Accident and Emergency, one of which ended up with a few nights on a hospital ward ensued. The portrayal of ‘you’re sick for a few weeks, then you’re okay’ or ‘you’re sick and you need Intensive Care’ just didn’t fit with my experience. It was difficult coping with the random patterns of symptoms, one day, *palpitations at night*, *to the severity that it felt like I’d almost jumped awake and leapt* off the bed; the next, *my fingers would go blue*, *and my feet*, the next, debilitating *headaches*, I could go on… *I felt like I just wasn’t being believed*, *like I was just*, *you know*, *making stuff up*. From what I have seen on online forums, I know that not everyone has had this experience, but I think they were in the minority, and getting any useful healthcare input was a bit of a ‘lottery’. Since then, I’ve made some progress, but I’m afraid to push it because of what happened last time, *I don’t want to have another setback*. I’m worried that I might end up developing a long-term condition; my GP is now talking about long-COVID, but nobody really knows how this will pan out either.

I do see myself as one of the lucky ones that at least managed to get a confirmed diagnosis, and that has provided me some comfort and reassurance that I’m not going mad. Other people seem to be a lot more understanding when you know for certain, but, on the other hand, when the numerous tests you’ve had keep repeatedly coming back clear, it still makes everything difficult to comprehend, and does nothing to help explain your ongoing symptoms. I constantly flip between feelings of despair, being fed-up and not wanting to be a burden, to trying to be optimistic that I’m not going to be like this forever. *I’m not looking for a quick fix… I want my recovery to last*.”

### Persistent—life-changing and lifelong… is this it now?

“It’s been a long haul, *the first few months kind of all blended into one*, and my journey doesn’t seem to be going to end any time soon. Initially, apart from just feeling *really dreadful*, I was scared. Scared about becoming critically unwell at home, *scared about the prospect of going into hospital*, scared about not knowing what was happening, and scared about spreading it to others. At one point, I thought I was going to die; I’ve even *written a will since then*… . *I was laid flat on the sofa all day because I didn’t have the energy to get up*… *There have been points when I’ve felt a bit worried in terms of like ‘am I ever going to recover from this*? *Is this ever going to change*? I’m so grateful that I had such a good support network, as at my worst I would have struggled to look after myself.

That acute phase may be over now, but the debilitating effects are ongoing. I’m still struggling to recover months later. It’s had, and still is having, a massive impact on my life. I’ve lost my role within my household, with my partner having to take on more, and *I wouldn’t have the energy* to return to work, to my *career*, even if I wanted to. I have to deal with the frustration of not being able to just do ‘normal’ activities without suffering the overwhelming malaise that comes after the exertion, and it’s not just after physical activity, it can be after mental exertion as well. And that brings me onto something that started later, the ‘brain fog’. *I couldn’t remember lots of words*. *I’d try and describe*… *I’d have to describe things I knew*. *Like a zebra crossing*, *I described it as a ‘stripy thing’*.

I have managed to get some relief from *realizing how many people were struggling with similar ongoing symptoms*, *weeks and weeks*, *or months*, *after being ill*. Social media groups have been a really good source of a lot of useful information, but only if you’re sensible with your access and try to avoid misinformation. Sometimes the negative comments could be quite distressing. Overall, *I’ve felt so traumatized by the whole thing*. *You lose an awful lot of control over what you can do*, *physically*, *mentally*, *and emotionally*, and often *there is a clash between my mind and my body*–the mind wants to, but the body can’t. *I’ve reached the point of desperation now where you’re willing to try anything* to help you recover, but I’m also really worried that this is it, and that *maybe the mindset that I need to have is to learn how best to manage living with these symptoms*, but I really hope that is not the case.”

## Discussion

The vignettes outlined represent the all-too-often unheard voices of those who have experienced COVID-19 and its aftereffects, especially those whose journey commenced long before we had any understanding of the trajectory it could take, or before strategies to facilitate recovery had started to be implemented. Whilst the experiences of the individuals portrayed by the vignettes cannot be changed, they present healthcare providers with the opportunity to learn from them, not just for individuals with a diagnosis of COVID-19, but also for others with multi-systemic medical conditions that can present in various different ways (i.e., Myalgic Encephalomyelitis [ME] and Chronic Fatigue Syndrome). Indeed, the lived experiences of individuals with ME varies significantly and the illness remains poorly understood [23], highlighting the need for such approaches to be adopted for other chronic conditions where many patients may currently be feeling under-represented, unheard, misunderstood and/or lacking in support due to the reliance on “one-size fits all” treatment and management strategies.

The first vignette, ‘Almost recovered—out of sight, out of mind, but I just had to get on with it’, highlights that even when symptoms were perceived to be relatively mild, the fear of the unknown, lack of knowledge surrounding the condition, and feeling that there was no help to access, still had long-term negative effects. Specifically, within this study, individuals, regardless of symptom severity, demonstrated reservations about trying to make that last step back to their prior lifestyle, ultimately being afraid to do so. These findings resonate with those of Wright et al. [24] where the provision of conflicting advice by healthcare professionals and potential attempts to avoid post-exertional symptoms were linked to hesitance in returning to pre-COVID physical activity levels. Failure to return to pre-COVID lifestyles and working behaviours has significant potential implications at individual, societal, and economic levels. [25]. Physiological and psychological parameters, changes to social and familial relationships, and changing financial and employment situations, all present challenges within the ‘survivorship’ of ongoing health conditions [26].

In the second vignette, *‘*Ongoing waves—trapped on the COVID roller-coaster, when the horizon just keeps falling out of sight’, the frustrations of repeatedly starting to recover and then coping with periodical setbacks often linked to a return to physical activity were highlighted. Although pacing, the limiting of physical and mental activities to stay within energy reserves to seek to avoid symptom exacerbation [24], was reported to help, risk adversity was again demonstrated. Whilst in accord with the standard recommendations for those recovering from COVID-19 [27], trading-off activities to manage prolonged symptoms (i.e., breathlessness and fatigue), often meant that social contact and participation in activities formally known as “normal life” became limited, subsequently negatively affecting mood levels and causing further anxiety. These findings contradict those of other studies where pacing was found to challenge avoidance behaviours and actually facilitate engagement with more activities [28] and increase PA levels [29] in a controlled way.

The third vignette, ‘Relapse—recover, relief, relapse, battling the unknown enemy’, presents an experience driven by an initial sense of relief regarding perceived recovery, only for this to subsequently be quashed, with individuals often thrown back to the beginning of their journey or down a different path. Similar to the findings of other research, individuals also depicted the struggle and indifference that came with being unable to explain or often justify their unpredictable and random symptom patterns to others [30]. The fourth, and final, vignette, ‘Persistent*—*life-changing and lifelong… is this it now?’ focused on those individuals whose COVID-19 experience is one of more consistent ongoing symptoms and despair. As previously noted, individuals struggled with adapting to changing dynamics and responsibilities within social and family contexts [5, 6]. Moreover, unease extended to not only short-term financial worries, but also the potential long-term implications on career status and progression opportunities.

Whilst the vignettes present four lived experience trajectories, it is pertinent to note that given the vast and varied presentation of symptoms (see Fig 1), the observed subjectivity of perceived severity and what is deemed to be a “relapse” or “exacerbation”, and the heterogeneity of long-COVID itself [31], some individuals may personally resonate with elements of more than one vignette. For instance, all participants discussed the difficulties of not being able to obtain a confirmed diagnosis. Whilst these difficulties initially related primarily to gaining access to healthcare and explaining their condition/experience to their family and employer, the secondary psychological and emotional effects of not knowing what they were dealing with, and what to do, could have profound implications on long-term health and well-being. Indeed, research suggests an association between persistent symptoms of a physical nature (i.e., breathlessness) and psychological health following discharge from hospital with COVID-19 [32], as well as the time since acute COVID-19 being associated with poorer psychological health [14]. Furthermore, as the understanding of COVID-19 has developed, it has become more apparent that post-COVID-19 syndrome is associated with impaired cognitive function, even in those with relatively mild acute presentation [33].

It is acknowledged that even if overall experience patterns are less dominant, or symptoms less frequent, this does not make them less important or signify their level of impact [3]. The acute limitations of having a cough or change in senses, although a hindrance, do not cause the same level of long-term physical or psychological concern, as for example, cardiac or neurological symptoms. However, understanding the most common presentations will not only forewarn other individuals about the potential trajectories they may experience, but also help healthcare providers to develop and prioritize the most effective use of resources, for the greatest amount of people. Moreover, a systematic review of experiences of accessing healthcare services for long-COVID has highlighted the need to account for patient perspectives when adapting or designing such services [31].

### Strengths, limitations and future research

Listening to and acknowledging experiences is an essential part of the recovery process, on both an individual and societal level. Indeed, the importance of being able to resonate with the experiences of others has been identified [34]. Within this study, the rigorous processes employed throughout data collection and analysis, coupled with a data set that included a rich description of experiences, enabled the identification of multiple distinct profiles.

Whilst there are numerous strengths, it is important to acknowledge limitations. Specifically, the sample was more representative of females (85%), which, given that it was not deemed possible to separate the experiences according to sex during data analysis, may mean that the composed vignettes more strongly represent the experiences of females than males. However, it is pertinent to note that the proportion of females included in this study is commensurate with those who experience long-term symptoms (three times greater risk) [35]. It is also noteworthy that a relatively high number of participants had a healthcare, or health and fitness background (51%) and were classified as highly or moderately active prior to the COVID-19 pandemic, potentially introducing an element of sample bias. Healthcare workers may have also experienced the pandemic differently from the general population, with some viewing infection as ‘*inevitable’* and having to cope with this fear [36]. Participants may have been driven to volunteer for the study based on either their background knowledge or pursuit of pre-COVID physical activity levels. Moreover, only a limited number of the study sample had a confirmed COVID-19 diagnosis. However, given that the majority of participants were infected during the initial phase of the pandemic, when testing availability was extremely limited, a confirmed positive test was not a pre-requisite for participation.

Key demographic data, including gender and work status were collected, or where possible extracted, from the interview transcripts, however, additional information, such as age, socioeconomic status, ethnicity, and pre-COVID mental and physical health, would have provided further context within both the analysis undertaken and in comparisons with future, similar studies. Therefore collection of such data should be considered in future research. At the research inception point, long-COVID, and the impact it could have on individuals was not widely recognized, or defined, therefore making it difficult in this instance, to incorporate patient and public involvement into the research design process. Future studies, exploring similar concepts should embrace this practice. Finally, given the long-term variability of symptom presentation, duration, and experience trajectory, there is a need for robust studies that: i) report longitudinal follow-up data; ii) explore the impact of contracting COVID-19 on repeated occasions; and iii) establish at-risk sub-groups and their specific support needs.

## Conclusion

The aim of creating these composite vignettes was to highlight and portray the day-to-day non-biological effects and wider ramifications that contracting and living with COVID-19 had on individuals during the early stages of the pandemic, from the point of their symptom onset through the course of their acute and ongoing long-term pursuit of recovery. The vignettes highlight: i) the potential negative implications of not addressing the psychological effects of COVID-19, which may contribute to the impact of ongoing physical symptoms [32]; ii) the lack of linearity not only in symptom development but also the trajectory of chasing recovery—the unpredictability and variability of persistent, fluctuating, relapsing, or new symptoms present a variety of challenges that can shape an individual’s experience in a multitude of different ways [11]; and iii) the ongoing ‘lottery’ of access to healthcare services and the secondary impact on individuals’ lives. Therefore, in conclusion, this novel study presents the lived experience of COVID-19 and consequent long-COVID in participants’ own words, revealing the highly variable, but generally devastating, impacts COVID-19 has had across many facets of daily living. Taken together, this research provides an insight into different experience trajectories which may enable more individually tailored treatments, on a population level.

## Supporting information

S1 File Standards for Reporting Qualitative Research: A synthesis of recommendations (SRQR) checklist. (PDF)

S2 File Exemplar of data synthesis process. (PDF)
