# Supplementary material for: Battling the unknown: Using composite vignettes to portray lived experiences of COVID-19 and long-COVID
Source: PLoS One. 2023 Apr 26;18(4):e0284710. doi: 10.1371/journal.pone.0284710 (PMC10132598; doi:10.1371/journal.pone.0284710)
Supplement: S2 File — (PDF) [file pone.0284710.s002.pdf]

S2 File

Example excerpts of initial manual coding and experience summary – Relapse

it again, because at the time, nobody knew about long Covid and then I just went on this complete downward spiral of virtually all the symptoms returned, except the temperature, so I was back to tachycardia, severe shortness of breath, this feeling that I could not expand my lungs, that, you know, that, that the lungs were made of concrete and just weren't budging and so there was nothing that anyone could really offer, the GP tried me on different inhalers, essentially the message was just rest and I'd have maybe five days of feeling absolutely awful and then I'd pick up and think I was coming out of it and then, you know, that might be for about three or four days and then wallop, terrible again. I haven't mentioned the fatigue but absolutely exhausted. So I could manage [illegible]

– Relapse
Message was – Rest

was probably about twelve hours behind me, so I'm assuming, I guess, I probably caught it from school and infected him, I guess

And when you say worried, what was it specifically you were worried about?

I was worried about, I was worried about having a heart attack, I was worried about being, being worse, I was worried about being hospitalised, I was worried about sort of never being quite normal again really, never being like the same again and I think that, that worry has, you know, that's sort of stayed with me, I'm not, I'm not a worryty person, I'm not a sort of an, desperately anxious person or a person that gets depression or anything like that, I'm a, I'm a bit of a coper really but I think it was because it felt so very different to anything else because I couldn't get any answers, nobody knew, you know, I spoke to a variety of GPs that, the best one was honest and said, look, we haven't, we really don't know but let's do this, this and the other, I had other GPs at the same surgery who, well one of them was one of those that sent me to, to the, the unit at

– Worried
Long term damage
& Worried will never be the same again
The Impact of the

[illegible] study, I had to have some evidence, so I, I paid for a Bupa test and I was, had positive antibodies in August. Where we are now, I've tried pacing, so I walk every day for about a mile and a half, it's eight months now since the original infection and I am starting to feel a little bit better actually, the, the weird feeling that I can, I, I, it was very hard to describe to people what the weird feeling was but on really bad days, it was like I'd been poisoned, it was like a really awful hangover kind of feeling and the, the weird feeling, I haven't had that since September the twenty eighth, my chest is still tight, I do feel a little bit short of breath when I run upstairs, try and run upstairs, and my walking pace is slower, I used to be a really fast walker and obviously I, I'm not even attempting to, to run, jog or anything remotely like that, I'm doing yoga and walking and that's sort of it really, I think, that's the overview

+ve Antibodies
Confirmed diagnosis
– Weird feelings

Yeah

I had, I had two, I, I'd had a couple of weeks I was really fluey and, and horrible and, and, you know, and as I said, I had two paramedic, well paramedic call out, two 111 calls and then I felt like I'd recovered and then, and then it was the, and then it was the crash but I, I did keep a Covid diary up to a certain point, to try and see a pattern, there was none, absolutely none whatsoever, there was no relationship to what I did, although if I had a good day and I did a little bit too much, I was probably worse the following day but that was before I discovered the, the pacing idea but there was no, no real pattern, you know, it could be two, two days good, five days rubbish or vice versa

And how long was that fluctuation going on for, of good days and bad days, was?

#4 → Symptoms linked to over exertion

And can you just describe how this has impacted your, your daily activities over the course of these months?

Impact on life.

Oh it's been huge, I mean I've given up a job I love, which actually does make me feel a bit emotional, I'd, you know, I was, I was doing part time [redacted] where I've taught for seventeen years, and, you know, I had no intention of, of stopping doing that, it's a job I absolutely loved and so that, you know, to, to, to be getting so little help or know where you're going that you feel that you've got to chuck your job in, I think that, that says it all. So that's massive. I, obviously I can't exercise like I did, I'm doing what I can and I do, you know, I'm enjoying my yoga and do it on Zoom here but I can't run and I'm seeing people running in the road because a lot of people were taking up running and that's, that's, you know, that, that does make me feel rather sad actually. [redacted] we will, we will go to Tescos together, he doesn't, he doesn't mind that and actually, when I was working, he used to go but, you know, I think doing the full shop and lugging the bags probably, you know, I wouldn't do that, I wouldn't be able to hoover the staircase, socially, social, the, something that just absolutely drains me is talking to people and obviously, as a [redacted] was talking all day long. I would, I have seen friends, I've say two hours is about my limit and that's even with, that's even with family, I've got three grown up children, who have got partners, and just the, the effort of socialising, so that, that's had the biggest impact. I haven't, I have turned down invites but also I have gone to things previous, you know, between lockdowns and people are very good actually and they'll often start it in the evening thing early and I probably have to leave about nine o'clock

Talking to people is draining

Okay

So I manage from say seven til nine and then I just have to go to bed because I'm, I'm absolutely wiped out with it

ID:	Sex:

HPC: (confirmed diagnosis/hospitalised/ healthcare input etc)
Managed at home
Rang 111 – reassured
111 due to heart – 999 – ECG – not sick enough for hospital – could have gone but chose not to.
Returned to walking everyday
Then relapsed
Positive antibodies

Initial Symptoms/ Concerns
Temperature/Flu symptoms/headaches/SOB/palpitations/upset stomach

Secondary Events/Relapse:
Complete downward spiral – all symptoms re-started; lungs felt like made of concrete

Relevant PMH/Underlying Conditions:
Allergic Asthma

Social Info:
Secondary school teacher

Previous Ex level / PA:
Very active Pre-COVID -run/gym/ etc

Management Input / Strategies:
GP input, inhalers etc, tests NAD
Yoga, Walking, Online groups, Prioritising, Pacing – much better once discovered the 'pacing idea'

Current Status

MLF
Fatigue

Work
Early retirement

Exercise /PA
Can't run

Overall
Acutely unwell, improved, relapsed, significant bouts of ongoing symptoms but improving fluctuations less dramatic

Emotions
Only went to hospital if on deaths door
Impact on/Altered life – took early retirement
Relief to get a confirmed diagnosis
On bad days, weird feeling, feel like been poisoned
Difficult as sometimes even small tasks can bring on symptoms
Worried about prognosis, being hospitalised, never being quite normal again really, never being the same
Anxious about virus causing long-term damage
Felt so different to anything else because can't get any answers
Frustration at lack of diagnosis, so paid for antibody test, felt vindicated confirmed diagnosis
Good to be able to communicate with people with same experiences (importance of peer support)
Difficult being dismissed by GP/having symptoms dismissed as being anxious, you've been watching the news too much....all in your head.
Cross with medical profession (but doesn't say why)
Find social situations draining – it's an effort so turns them down
Life now a complete reversal of pre-COVID
Just wants to be back where they were, difficult not being able to do pre-covid activities – sees a runner – oh look I wish that was me

Initial summation of experiences into vignettes

**Experience Trajectory -**	**Participant Number**
Acutely unwell	n = 46
Slow gradual Improvement	2,4,6,7,8,40,42,46
Developed additional secondary symptoms	4,
Full recovery	
Still not back to baseline, limited by ongoing symptoms	2,8,40,42,46
Symptoms now nearly fully resolved	4,7
Prolonged ongoing symptoms	6
Initial Improvement then relapse	1,24,27,28,29,31,33,41,51,54,58,59,61,74,79,80
Developed additional secondary symptoms	24,27,29,31,33,58,59,61,79,80
Ongoing symptoms that are gradually improving	59,80
Ongoing waves of symptoms that are improving	1,74,79
Ongoing waves of limiting symptoms	28,31,33,51,61
Ongoing persistent/debilitating symptoms	1,23,24,27,29,39,41,54
Probable long-covid	1,23,27,28,29,31,41,59,61,74
Then improved gradually now plateau'd	58
Initial improvement but ongoing waves of symptoms	5,10,12,15,16,17,25,35,36,37,38,44,50,55,64,66,68,75,76,77
Gradually decreased in severity and frequency	5,12,15,35,37,44,76
Flare up when tests themselves	16,25,36,37,64,77
Still debilitating	
Probable Long-Covid	5,10,12,15,16,17,25,35,36,37,38,44,50,55,64,66,76,77
Recovery plateau'd	10,55
Developed additional secondary symptoms	50,64,66,75,77
Incidental finding, retrospective awareness of COVID	11
Late onset sporadic symptoms, now back to normal	11

Revised summation of experiences and finalisation of four outline vignettes

Experience Trajectory -	Participant number
Acutely unwell	n = 46
Incidental finding -	11
*Initial Improvement then **relapse***	5,15,23,24,27,28,29,31,33,39,51,54,58,59,61,74,80
Developed additional secondary symptoms	15,24,27,29,31,33,58,59,61,80
Ongoing symptoms that are gradually improving but still debilitating	15,39,54,58,59,80
Ongoing waves of symptoms that are improving, but still limiting	51,74,
Ongoing waves of limiting symptoms	28,31,33
Ongoing persistent/debilitating symptoms	23,24,27,29,61
Probable long-covid	23,24,27,28,29,31,51,54,59,61,74
*Initially some improvement but **ongoing waves of symptoms***	12,16,17,38,40,44,50,68,79
Gradually decreased in severity and frequency/still present but milder	12,40,44,68,79
Flare up when tests themselves	16,79
Still debilitating	
Probable Long-Covid	12,16,17,38,40,44,50,68,79
Recovery plateau'd	
Developed additional secondary symptoms	50,
Incidental finding, retrospective awareness of COVID	11
Late onset sporadic symptoms, now back to normal	11
Milder pattern of Initial symptoms - Gradual recovery some up & downs /lingering or secondary symptoms but reasonably straight forward, **almost recovered** but can't quite get back to baseline	2,4,7,8,42,76
*Acutely unwell, prolonged ongoing symptoms, little improvement - worse with increased activity - **persistent***	1,6,10,25,35,55,64,75,77
Developed additional secondary symptoms	1,6,35,64,75,77
*Acutely unwell, slowly improving, but still limited by **persistent** symptoms*	36,37,41,46,66
